# *Parvicapsula pseudobranchicola* in the northeast Pacific Ocean is rare in farmed Atlantic salmon *Salmo salar* despite widespread occurrence and pathology in wild Pacific salmon *Oncorhynchus* spp.

**DOI:** 10.1186/s13071-023-05751-y

**Published:** 2023-04-21

**Authors:** Simon R. M. Jones, Jessica C. Low, Aidan Goodall

**Affiliations:** Fisheries and Oceans Canada, Pacific Biological Station, Nanaimo, BC Canada

**Keywords:** Marine myxozoan, *Oncorhynchus* spp., Pacific, Canada, Quantitative polymerase chain reaction, In situ hybridisation

## Abstract

**Background:**

Infection with the myxozoan parasite *Parvicapsula pseudobranchicola* causes disease in wild and farmed salmonids in Norway. In the northeast Pacific Ocean, the parasite has been reported in Pacific salmon *Oncorhynchus* spp. without evidence of disease. The objectives of the present study were to confirm the identity of *P. pseudobranchicola* in the Pacific, document its host and geographic ranges, and describe associated pathological changes.

**Methods:**

Ocean-entry year wild pink salmon *Oncorhynchus gorbuscha*, chum salmon *O. keta*, Chinook salmon *O. tshawytscha*, coho salmon *O. kisutch* and sockeye salmon *O. nerka* were collected in summer and autumn surveys near Vancouver Island (VI) and from a winter survey in the Gulf of Alaska. Samples were also obtained from farmed Atlantic salmon *Salmo salar* and Chinook salmon near VI. Samples were analysed by qPCR and histology using conventional staining or in situ hybridisation. Parasite sequence was obtained from small subunit ribosomal RNA gene (SSU rDNA).

**Results:**

Identical 1525 base-pair SSU rDNA sequences from infected pink salmon, chum salmon and Chinook salmon shared 99.93% identity with a *P. pseudobranchicola* sequence from Norwegian Atlantic salmon. In autumn surveys, the prevalence was greatest in chum salmon (91.8%) and pink salmon (85.9%) and less so in Chinook salmon (68.8%) and sockeye salmon (8.3%). In farmed salmon, the prevalence was zero in Atlantic salmon (*n* = 967) and 41% in Chinook salmon (*n* = 118). Infections were preferentially sited in pseudobranch and visualised by in situ hybridisation. Heavy parasite burdens in all species of Pacific salmon were inconsistently associated with focal granulomatous pseudobranchitis.

**Conclusions:**

In the northeast Pacific, widespread occurrence of *P. pseudobranchicola* in Pacific salmon together with its absence or sporadic occurrence in farmed Atlantic salmon differs from its epidemiology in Norway, despite similar pathological development in the pseudobranch. Consequences of the infections to the health of wild Pacific salmon, identity of the invertebrate host and the distribution and abundance of infective actinospores are unknown and remain high priorities for research.

**Graphical Abstract:**

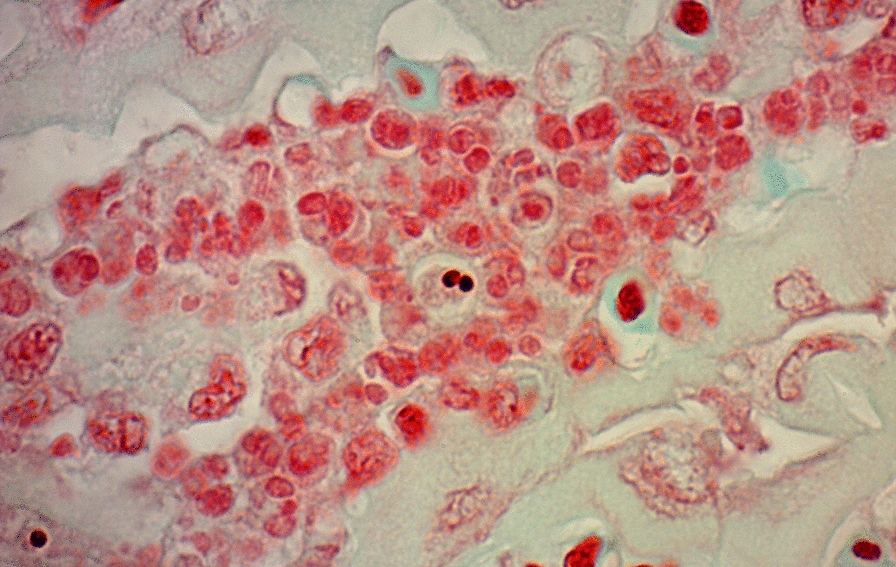

**Supplementary Information:**

The online version contains supplementary material available at 10.1186/s13071-023-05751-y.

## Background

Members of the genus *Parvicapsula* (Cnidaria, Myxosporea) are parasites of the urinary bladder, gall bladder, intestinal epithelium and pseudobranch in marine or anadromous teleosts in which they have occasionally been shown to cause disease [1, 2]. Of the 16 known *Parvicapsula* spp., three have been reported from salmonid hosts. In western North America, *Parvicapsula kabatai* [3, 4] and *P. minibicornis* [5–11] are parasites of anadromous Pacific salmon *Oncorhynchus* spp. *Parvicapsula kabatai* was detected by using molecular methods in farmed coho salmon *Oncorhynchus kisutch* in Washington State (USA) [3], previously found to be infected with *Parvicapsula* sp. [12]. With the exception of *P. minibicornis* whose invertebrate host is the freshwater polychaete *Manayunkia occidentalis* [13], the life cycles of *Parvicapsula* spp. are unknown.

*Parvicapsula pseudobranchicola* was originally described in northern Norway from marine farmed Atlantic salmon *Salmo salar* [14] in which it causes inflammation and necrosis of the pseudobranch as well as the non-specific clinical signs of lethargy, anorexia and dark pigmentation (Table 1) [14–16]. The parasite has also been reported from wild Atlantic salmon, farmed rainbow trout *Oncorhynchus mykiss* and wild sea trout *Salmo trutta* in southern and northern Norwegian coastal waters [17–20]. In the northeastern Pacific waters of British Columbia (BC), Canada, *P. pseudobranchicola* has been detected in adult sockeye salmon *Oncorhynchus nerka*, coho salmon and Chinook salmon *O. tshawytscha* [21–24] and in juvenile Chinook salmon and coho salmon [25]. In BC, the parasite was detected in farmed Atlantic and Chinook salmon [26]. The parasite was also detected in coho salmon, sockeye salmon, chum salmon *Oncorhynchus keta* and pink salmon *O. gorbuscha* overwintering in international waters of the Gulf of Alaska [27]. The Pacific detections of *P. pseudobranchicola* are based on molecular evidence with neither microscopic observations of the parasite nor evidence of disease caused by the infection.

**Table 1:** Host, site of infection and location of *Parvicapsula pseudobranchicola*

Host (common name)	Site	Location	Refs.
*Salmo salar* (Atlantic salmon) *S. trutta* (sea trout) *Salvelinus alpinus* (Arctic charr) *Oncorhynchus mykiss* (rainbow trout)	Pseudobranch, gill, liver, kidney	Norway^a^: north, west and south coasts	[14, 15, 17–20]
*S. salar* *O. nerka* (sockeye salmon) *O. kisutch* (coho salmon) *O. tshawytscha* (Chinook salmon) *O. keta* (chum salmon) *O. gorbuscha* (pink salmon)	Liver, tissue homogenates	Pacific Ocean^b^: British Columbia, Gulf of Alaska	[21–27]

^a^Detection by microscopy, molecular and/or clinical methods

^b^Detection by molecular methods

The objectives of the present study were to use PCR and sequence data together with site preference and morphology to confirm the identity of the parasite presently described as *P. pseudobranchicola* in BC, to document its host range and geographic distribution and to describe pathological changes associated with the infection.

## Methods

### Fish collections and tissue samples

**Fig. 1:**
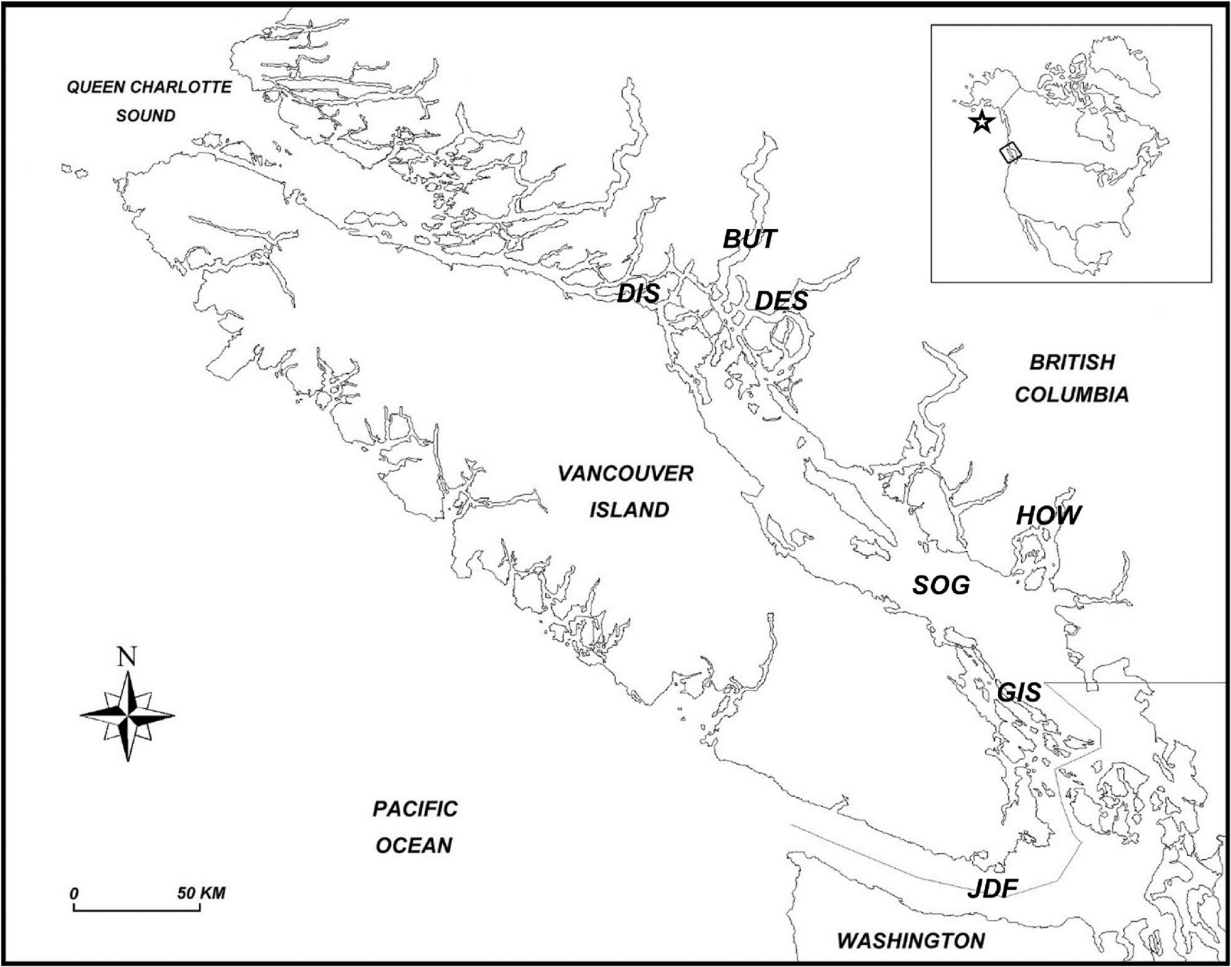
Map showing Pacific waters adjacent to Vancouver Island, British Columbia, Canada, with areas from which wild Pacific salmon (*Oncorhynchus* spp.) were collected. DIS, Discovery Islands; BUT, Bute Inlet; DES, Desolation Sound; SOG, Strait of Georgia; HOW, Howe Sound; GIS, Gulf Islands; JDF, Strait of Juan de Fuca. Star in inset approximates the Gulf of Alaska

Chum salmon, pink salmon, coho salmon, sockeye salmon and Chinook salmon were caught by trawl and purse seine gear in summer and/or autumn surveys in BC waters in 2008, 2010–2015 and 2019–2021 (Additional file 1: Table S1). The fish were collected from areas adjacent to Vancouver Island including Discovery Islands, Bute Inlet, Desolation Sound, Strait of Georgia, Gulf Islands, Howe Sound and the Strait of Juan de Fuca (Fig. 1). Chum salmon, pink salmon and coho salmon were also collected in a winter survey from international waters in the Gulf of Alaska in 2020 (Additional file 1: Table S1).

The 306 fish caught in 2008 and 2010–2015 and the 133 caught in the Gulf of Alaska were frozen immediately after capture and later thawed in the laboratory, weighed and processed. The 1249 salmon collected in 2019–2021 were identified, weighed and tissue samples preserved within 2 h of capture. Pseudobranch samples from all fish and individually matched mid-kidney and/or gill samples from subsets of fish were aseptically dissected and preserved in 95% ethanol. Of the 1249 salmon collected in 2019 to 2021, replicate tissue samples from 1218 were preserved in 10% neutral-buffered formalin (NBF). Pseudobranch samples from farmed Atlantic and Chinook salmon collected in 2019 and 2020 and preserved in RNAlater were provided by the Fisheries and Oceans Canada’s Fish Health Audit and Intelligence Program (DFO-FHAIP). The Atlantic salmon were collected during scheduled audits from aquaculture facilities within the fish health zones (FHZ) 2.3 (Clayoquot Sound), 2.4 (Nootka Sound and Quatsino Sound), 3.1 (Sechelt), 3.2 (Discovery Islands), 3.3 (Broughton Archipelago), 3.4 (Port Hardy) and 3.5 (Central Coast) (Aquaculture maps Pacific Region (dfo-mpo.gc.ca)). The Chinook salmon were collected from FHZ 2.3 and 3.2.

### DNA extraction and qPCR

DNA was extracted from the ethanol- and RNAlater-preserved tissues and from previously frozen tissues by using the DNeasy^®^ Blood and Tissue kit (Qiagen). The DNA concentration was quantified by absorbance at 260 nm and samples then stored at − 20 °C until analysis. The qPCR assay was performed using primer and probe sequences targeting a 187-bp fragment of the *P. pseudobranchicola* 18S rRNA gene [18]. An individual reaction consisted of 1X TaqMan^™^ Universal PCR Master Mix (Applied Biosystems), 400 nM of the primers Parvi2fwd and Parvi1rev, 200 nM of the Parviprobe (Table 2), 5 μl DNA template and nuclease-free water for a final reaction volume of 25 μl. Each sample was screened in triplicate on the CFX96 Touch Real-Time PCR detection system (Bio-Rad) or StepOnePlus Real-Time PCR detection system (Applied Biosystems). Extractions and amplifications followed manufacturers’ protocols.

**Table 2:** Nucleotide primers and probes used for qPCR (a), PCR and sequencing (s) and in situ hybridisation (i) assessments of *Parvicapsula pseudobranchicola* small sub-unit DNA

Name (purpose)	5ʹ–3ʹ sequence	Ref.^a^
PCF7 (s)	SGAWAGTTTGATCGAATTTCTGCC	[19]
PCR5 (s)	AACACGCAGTTGGTGACTCG	[19]
PCF1 (s)	AAACTCAAGTTTTCGGGTTACGG	[19]
PCR5 (s)	AACACGCAGTTGGTGACTCG	[19]
PCF4 (s)	CTGTGTAAGTTCTTTCCGACATGG	[19]
PCR3 (s)	AACTAGACACCGTGGTCTCGCTCG	[19]
Myxgen4R (s)	ACCTGTTATTGCCACGCT	[28]
MX5 (s)	CTGCGGACGGCTCAGTAAATCAGT	[29]
LIN3F (s)	GCGGTAATTCCAGCTCCA	[30]
MYX1f (s)	GTGAGACTGCGGACGGCTCAG	[31]
PPSE.R (s)	AAAAACCGACGGTAGCACAC	PS
Parvi2fwd (a)	CAGCTCCAGTAGTGTATTTCA	[18]
Parvi1rev (a)	TTGAGCACTCTGCTTTATTCAA	[18]
Parviprobe (a)	FAM-CGTATTGCTGTCTTGACATGCAGT-Eclipse	[18]
Parvi_LNA (i)	DIG-TGTCAAAGACAGCAATACGG-DIG	[32]

^a^*PS* present study

For each qPCR run, the number of copies of target sequence per reaction (c/rxn) was determined from standard dilutions of a *P. pseudobranchicola* target sequence (gBlock, IDT Technologies) [33] and normalised to c/ng DNA. The limit of detection (LOD) was determined from a series of tenfold dilutions of the gBlock from 1000 to 15.625 copies per µl spiked into a pink salmon gill homogenate and extracted (Qiagen DNeasy^®^ Blood and Tissue kit) assuming 80% DNA recovery. The LOD was estimated as the lowest DNA concentration from which a Ct value was obtained in ≥ 50% of six replicates [34]. Only reactions ≥ LOD (10 c/rxn) were reported as positive.

### Sequencing

Parasite DNA samples from the most heavily infected pink salmon, Chinook salmon and chum salmon were amplified by PCR (Table 2) and reaction products were purified with ExoSAP-IT (Thermo Fisher Scientific). The sequences obtained using Sanger technology (Génome Québec) were edited and assembled using Sequencher 5.1, archived in Genbank and analysed by BLAST (http://blast.ncbi.nlm.nih.gov/Blast.cgi).

### Histology

NBF-fixed pseudobranch tissues were stored in 95% isopropanol, then dehydrated in an alcohol gradient, clarified in xylene, infiltrated with paraffin wax and sectioned for routine histological examination. Histological sections of pseudobranch from pink salmon (*n* = 5), chum salmon (*n* = 5) and Chinook salmon (*n* = 3) with no molecular evidence of infection and from those with the highest c/rxn (*n* = 9, 8 and 11, respectively) were stained with haematoxylin and eosin, Giemsa or Gram-Twort stains and were processed for in situ hybridisation (ISH) and examined by light microscopy. An earlier ISH protocol [35] was followed using 1 μM of the Parvi LNA digoxigenin-labelled probe [32] (Table 2), without acetylation and with 0.5% Light Green SF Yellowish counterstaining. Semi-quantitative histopathology and ISH scores were adopted based on extent of damage and number of stained parasites, respectively (Additional file 1: Table S2).

### Statistical analysis

Weight and parasite burden (c/ng DNA) data were not normally distributed, and non-parametric tests were used. Differences in median fish weights and median parasite burdens between summer and autumn collections were tested using the Mann-Whitney (MW) test. Comparisons of median burdens among species were tested using the Kruskal-Wallis (KW) test, with pairwise multiple comparisons tested using Dunn’s method (Sigma Plot 13.0). The significance of differences in prevalence was tested using the Chi-square test. Results of all tests were considered statistically significant if *P* ≤ 0.05.

## Results

### Fish data

#### Wild salmon

All salmon examined were in their first year at sea. In BC and with the exception of one coho salmon (56.0 g), the median weights of chum salmon, pink salmon, Chinook salmon and sockeye salmon collected during summer surveys were ≤ 26.5 g (Additional file 1: Table S1). The median weights of chum salmon, pink salmon and sockeye salmon collected in autumn surveys were significantly larger than those collected in summer surveys the same year (Additional file 1: Table S1). The chum salmon, pink salmon and coho salmon collected from the Gulf of Alaska (GOA) were first sea-winter fish and correspondingly heavier than the fish collected in summer (Additional file 1: Table S1).

#### Farmed salmon

A total of 967 Atlantic salmon were sampled during 148 audits. The mean number of days following transfer to sea (dps) of the audited salmon was 329.8 (range 22–740). Of these, 199 salmon were audited between 22 and 150 dps. A total of 118 Chinook salmon were sampled during 16 audits, with a mean of 336.1 (1–812) dps. Of these, 27 Chinook salmon were audited between one and 150 dps.

### Parasite prevalence

#### Wild salmon

In BC waters, *P. pseudobranchicola* was detected in four of the five species of Pacific salmon examined. In 2019 and 2021, the prevalence in pink salmon and chum salmon was significantly higher in autumn compared with summer surveys (Table 3), and this pattern was observed in multiple regions (Additional file 1: Table S3). In autumn surveys from all years, prevalence of the parasite was greatest in chum salmon (91.8%) and pink salmon (85.9%) and less so in Chinook salmon (68.8%) and sockeye salmon (8.3%). None of 114 fish collected between 2008 and 2011 was infected, and nine of 189 (4.8%) collected between 2012 and 2015 were infected. However, between 2019 and 2021, 808 of 1382 fish (58.4%) tested positive for the infection, reflecting the inclusion of more fish from autumn surveys in the latter years (Table 3). The parasite was undetected in 32 coho salmon, 31 of which were from the GOA. Overall, two chum salmon out of all 133 salmon examined from the GOA were found to be infected (Table 3).

**Table 3:** Prevalence and burden of *Parvicapsula pseudobranchicola* in pseudobranch of wild Pacific salmon (*Oncorhynchus* spp.) from coastal waters of British Columbia (BC) and from international waters of the Gulf of Alaska

Species	Year	Survey		
		Winter^a^	Summer^b^	Autumn^c^
Chum	2008		(25, 0)^d^	
	2011		(30, 0)	
	2012		(11, 0)	(10, 20.0) 0.08, 0.26
	2013		(17, 0)	
	2014		(51, 0)	(8, 37.5) 0.04 (0.01–0.07)
	2015		(4, 0)	
	2019		(139, 18.0) 0.81 (0.16–4.57)	(177, 96.0: Χ^2^ = 200.77, *df* = 1, P < 0.001) 28.8 (8.40–87.55) (U-statistic = 441.0, P < 0.001)
	2020	(93, 2.2) 0.01, 0.07		(130, 95.4) 7.29 (1.18–21.32)
	2021		(182, 5.5) 0.21 (0.11–0.42)	(294, 97.6: Χ^2^ = 405.66, *df* = 1, P < 0.001) 18.5 (4.26–47.10) (U-statistic = 156.0, P < 0.001)
	Total^e^	2/93	35/459	586/619
Pink	2010		(59, 0)	
	2012		(28, 0)	
	2014		(50, 2) 0.04	(10, 30.0) 0.02 (0.02–0.13)
	2019		(6, 16.7) 0.11	(21, 61.9: Χ^2^ = 3.83, *df* = 1, P = 0.05) 7.15 (2.33–17.44)
	2020	(9, 0)		(109, 66.1) 2.28 (0.27–13.08)
	2021		(43, 11.6) 2.86 (0.63–6.97)	(43, 97.7: Χ^2^ = 64.2, *df* = 1, P < 0.001) 2.96 (0.57–6.68) (U-statistic = 104.0, P = 0.99)
	Total	0/9	7/186	130/183
Chinook	2019		(4, 0)	
	2021			(80, 68.8) 2.90 (0.43–10.71)
	Total		0/4	55/80
Coho	2019		(1, 0)	
	2020	(31, 0)		
	Total	0/31	0/1	
Sockeye	2019		(8, 12.5) 0.12	(12, 8.3) 9.67
	Total		1/8	1/12

^a^Gulf of Alaska, March

^b^BC, May to July

^c^BC, September and October

^d^(Number tested, % positive) median qPCR copy number per ngDNA (interquartile range). Individual copy numbers given when number of positive fish < 3

^e^Total number of fish infected/tested. Statistical significance of differences between summer and autumn surveys in prevalence (Chi-square test) or median copy number per reaction (Mann-Whitney test)

#### Farmed salmon

Of the 1085 salmon examined in 2019 and 2020, the parasite was detected in 48 of 118 (40.7%) Chinook salmon from two FHS zones. The mean days at sea for infected Chinook salmon was 415.7 (1–812). The overall prevalence in Chinook salmon farmed in zone 3.2 (73.9%) was greater than in zone 2.3 (32.6%) (Table 4) (Χ^2^ = 13.08, *df* = 1, P < 0.001). The parasite was not detected in any of 967 Atlantic salmon from seven zones (Table 4).

**Table 4:** Quantitative PCR prevalence of *Parvicapsula pseudobranchicola* in pseudobranch of farmed Atlantic salmon (*Salmo salar*) and Chinook salmon (*Oncorhynchus tchawytscha*) by fish health zones in British Columbia

Species	Year	Fish health zone^a^						
		2.3	2.4	3.1	3.2	3.3	3.4	3.5
Atlantic	2019	(97, 0)^b^	(108, 0)	(34, 0)	(64, 0)	(124, 0)	(46, 0)	(22, 0)
	2020	(78, 0)	(67, 0)	(19, 0)	(110, 0)	(97, 0)	(73, 0)	(28, 0)
	Total^c^	0/175	0/175	0/53	0/174	0/221	0/119	0/50
Chinook	2019	(53, 35.8) 0.21 (0.05–0.40)			(8, 100) 9.40 (7.58–71.15) (U-statistic=10.0, P < 0.001)^*^			
	2020	(42, 28.6) 0.47 (0.21–0.84)			(15, 60.0) 0.06 (0.04–5.77)			
	Total	31/95			17/23			

^a^2.3, Clayoquot Sound; 2.4. Nootka Sound, Quatsino Sound; 3.1, Sechelt; 3.2, Discovery Islands; 3.3, Broughton Archipelago; 3.4, Port Hardy; 3.5, Central Coast

^b^(Number tested, % positive) median qPCR copy number/ngDNA (interquartile range)

^c^Number of fish infected/tested

### Parasite burden

#### Wild salmon

In the 2019 and 2021 autumn surveys, parasite burden (median c/ng DNA) in pseudobranch of chum salmon were significantly higher than in summer surveys, whereas in pink salmon these seasonal differences were not statistically significant (Table 3). In the 2021 autumn survey, median c/ng DNA in chum salmon was higher than in Chinook salmon and pink salmon, whereas the difference between Chinook salmon and pink salmon was not statistically significant (H-statistic = 55.99, *df* = 2, P < 0.001). In the 2019 and 2020 autumn surveys, median c/ng DNA in chum salmon was higher than in pink salmon (U-statistic = 6976.0. P < 0.001).

#### Farmed salmon

In 2019 but not in 2020, median c/ng DNA was significantly higher in Chinook salmon from FHS zone 3.2 compared with zone 2.3 (Table 4).

### Site of infection

*Parvicapsula pseudobranchicola* was detected in individually matched pseudobranch, gill and kidney samples from chum salmon, pink salmon and Chinook salmon collected in the 2021 autumn survey. Among the three species, the infection was consistently detected in a higher proportion of pseudobranch samples, followed by gill and kidney (Additional file 1: Table S4). Similarly in all three species, the median parasite burden in pseudobranch was significantly higher than in gill or kidney (Additional file 1: Table S4). Burdens in gill and kidney differed significantly in chum salmon but not in Chinook salmon or pink salmon (Additional file 1: Table S4).

### Sequencing

Parasite SSU rDNA sequences obtained from infected pink salmon (GenBank accession no. OP133363), Chinook salmon (OP133361) and chum salmon (OP133362) were 1525 base pairs (bp), 1549 bp and 1565 bp in length, respectively, and were identical to one another. The Pacific sequence differed from the available *P. pseudobranchicola* sequence (AY308481), obtained from an infection in Atlantic salmon in northern Norway in 2003, with a single G—A substitution (position 1420, 99.93% identity).

### Histology

Histopathological changes were not observed in 13 pseudobranch samples which had tested negative for the infection. A range of histopathological lesion scores and ISH scores was observed among 28 samples from all three species with burdens ranging from 11.46 to 3410.98 c/ng DNA (Additional file 1: Table S1). Similar microscopic lesions were observed in infected Chinook salmon, chum salmon and pink salmon, the most mild of which included a diffuse interlamellar proliferation of fibrocyte-like cells between the basal or distal lamellae. An increase in the extent of organ involvement was associated with filament and lamellar degeneration and necrosis. Infiltration of the lesion with polymorphonuclear cells and lymphocytes was occasionally observed, as was the formation of focal granulomas. The typical lesion was a focal, granulomatous pseudobranchitis, the most extensive of which was observed in Chinook salmon. In Chinook salmon, pathology ranged from normal to extensive, and the three fish with the highest pathology scores occurred among the four highest ranked for burdens (Additional file 1: Table S1). Similarly, four of five Chinook salmon with the highest ISH scores occurred among the five highest ranked for burdens. In chum salmon, despite having the highest overall burdens, pathology scores were normal to moderate, and there was no obvious association with ISH score rankings. In pink salmon, three of the four highest ISH scores aligned with the highest burdens and there was no obvious correspondence between pathology and burden.

**Fig. 2:**
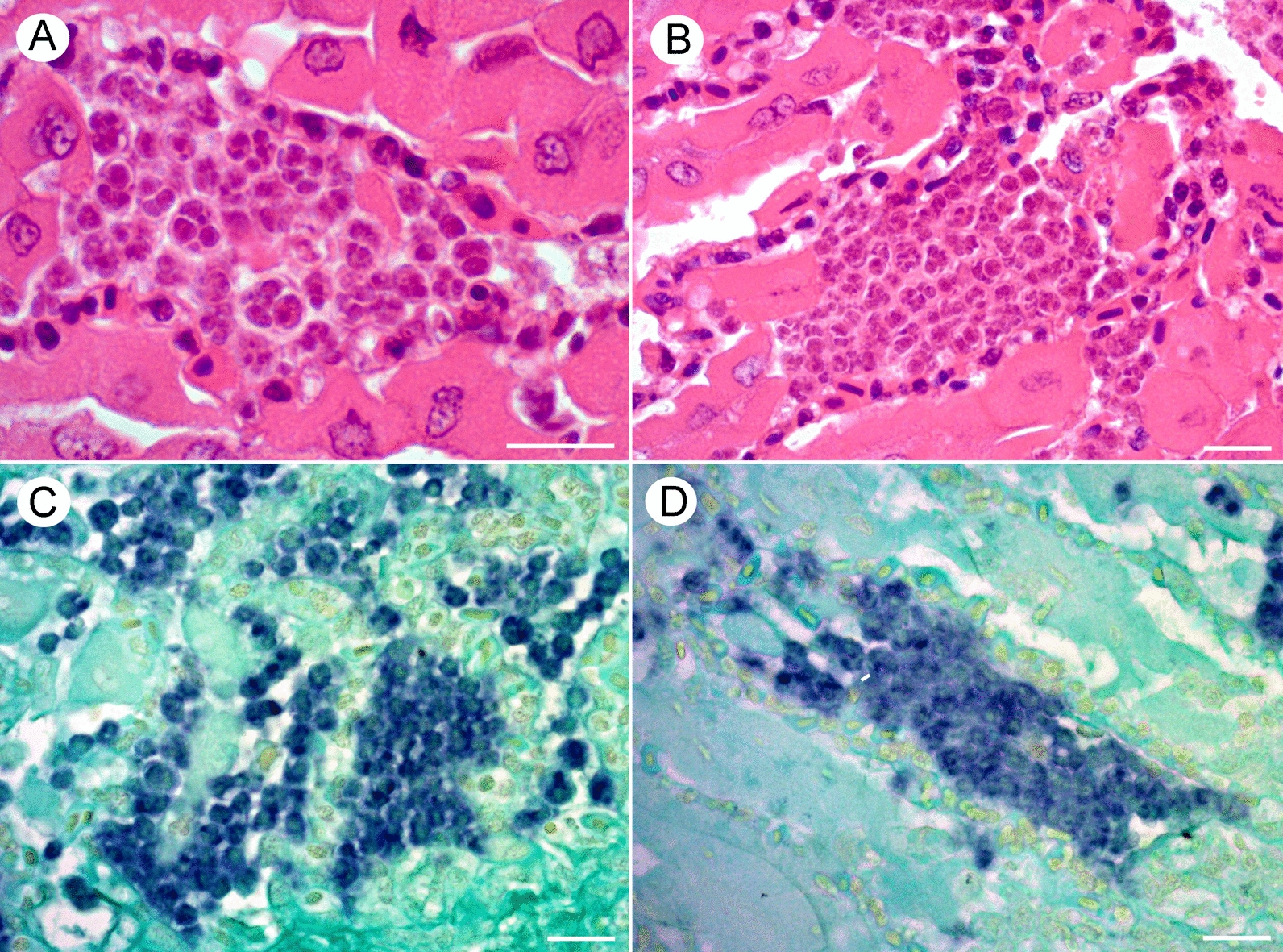
*Parvicapsula pseudobranchicola* within and between pseudobranch lamellae in histological preparations from juvenile Chinook salmon (*Oncorhynchus tshawytscha*). **A**, **B**: H&E staining. **C**, **D**: In situ hybridisation. Scale bars all 10 μm

**Fig. 3:**
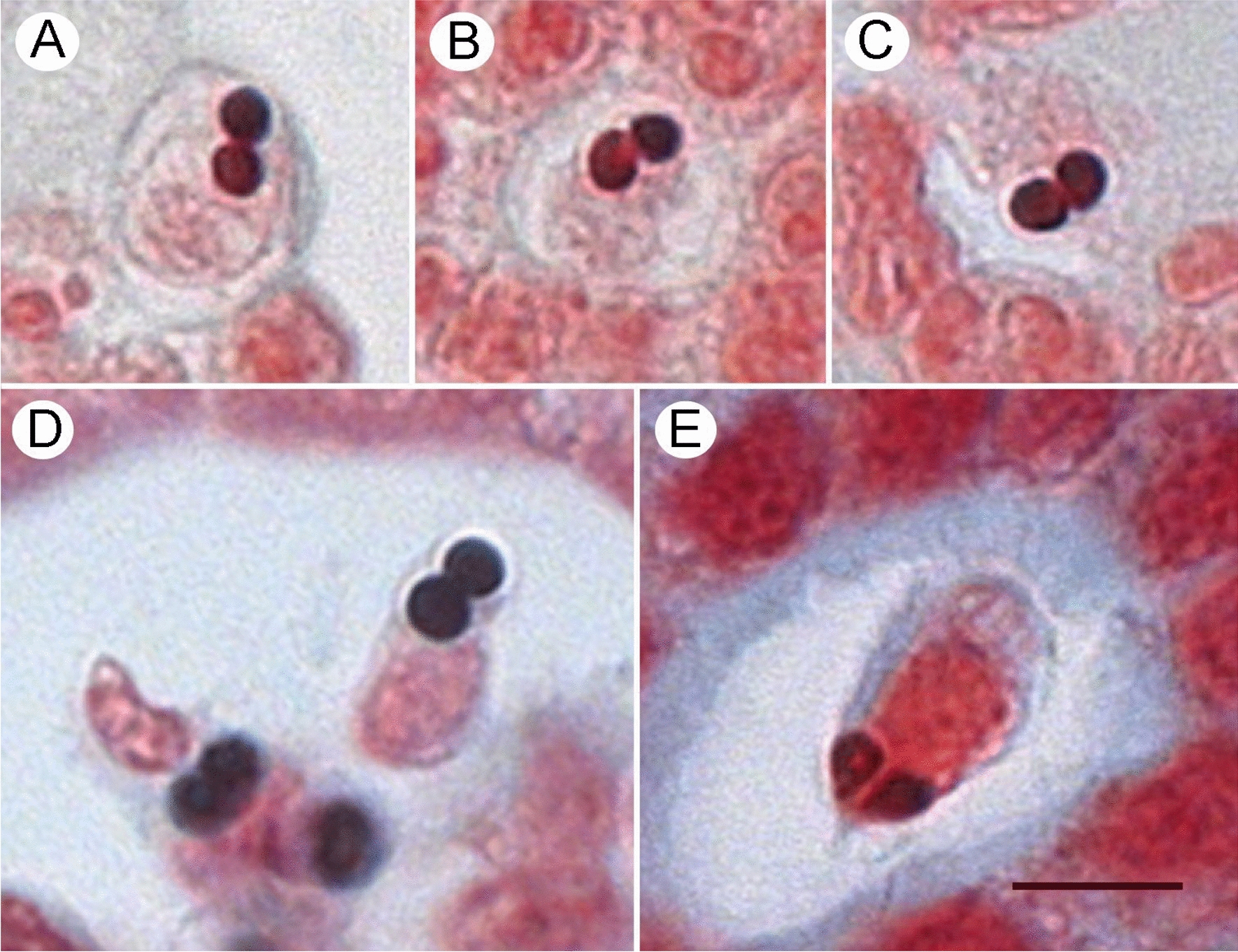
Histological preparations from Pacific salmon (*Oncorhynchus* spp.) in British Columbia, Canada, showing myxospores of *Parvicapsula* spp. **A**–**C** *Parvicapsula pseudobranchicola* in pseudobranch of juvenile Chinook salmon (*Oncorhynchus tshawytscha*) from the Strait of Georgia. **D** *P. kabatai* in renal tubule of adult pink salmon (*Oncorhynchus gorbuscha*) from the Quinsam River. **E** *Parvicapsula minibicornis* in renal tubule of sockeye salmon (*Oncorhynchus nerka*) from the Fraser River. Gram-Twort stain. Scale bar is 5 μm

Conventional histological staining permitted visualisation of the parasite in the two Chinook salmon with the highest burden (Fig. 2), which were the only fish with visible myxospores (Fig. 3). Myxospores were membrane-bound, and earlier developmental stages occurred as multi-cell clusters. Mean dimensions of myxospores (*n* = 5) were 5.8 × 4.8 μm. Polar capsules were subspherical and 1.7 μm in diameter. Fresh material was not available. In these and the remaining fish for which histological analysis was conducted, *P. pseudobranchicola* was unambiguously observed by ISH. In most infections detected by ISH, relatively few morphologically indistinct stages were observed with an apparent tendency to be distally located within pseudobranch filaments. The ISH probe did not bind to *P. minibicornis* or *P. kabatai*.

## Discussion

*Parvicapsula pseudobranchicola* was identified in Pacific salmon from Canadian and international waters of the northeast Pacific Ocean by demonstrating virtual identity between a SSU rDNA sequence from infections in chum salmon, pink salmon and Chinook salmon with that from an infected Atlantic salmon from northern Norway. The similarity between the Canadian and Norwegian sequences agrees with several earlier studies in which high intraspecific identities of SSU rDNA sequences were demonstrated among geographical isolates of other widespread myxozoan parasites [36–38], reflecting in part the relatively conserved region of the SSU rDNA targeted for amplification [39]. Large subunit (LSU) and internal transcribed spacer (ITS-1) rDNA sequences previously shown to be informative in resolving the phylogeography of *Kudoa thyrsites*, another cosmopolitan marine myxozoan parasite [37], may also be useful in characterising genetic heterogeneity among geographic isolates of *P. pseudobranchicola*. The present information, however, permitted unambiguous identification of the parasite in Pacific salmon. Although the myxospores observed here were superficially similar to their appearance in Norwegian cases, their relatively small mean length (5.8 μm versus 12.4 or 14.4 μm) [14, 15] may be related to immaturity, as indicated by their inclusion within the pseudoplasmodium membrane, in addition to artifactual changes associated with histological processing. In agreement with earlier indications of a broad host range in the northeast Pacific [21–24], *P. pseudobranchicola* was detected in nearly half of all juveniles examined belonging to four species of Pacific salmon. However, the parasite was most frequently detected in chum salmon and pink salmon. This broad host range is consistent with findings from Norway where the parasite has been reported in Atlantic salmon, sea trout, Arctic charr (*Salvelinus alpinus*) and rainbow trout [17–19].

In Norway, screening of Atlantic salmon pseudobranch throughout a farm production cycle initiated by transfer of smolts to sea in autumn revealed 100% prevalence of *P. pseudobranchicola* by 21 dps, followed by an increase in histologically evident parasites between 35 and 49 dps and the presence of myxospores by 89 and 147 dps [20]. At the site under study, the parasite burden declined sharply after 147 dps and there was no increase during the second autumn at sea [20]. Other studies from Norway report similarly high prevalence in farmed Atlantic salmon and found that among salmon transferred to sea in spring, infections were detected in July and spores were visible by autumn [17–19]. The elevated infections observed in the summer and autumn in Norway were suggested to be caused by an elevated abundance of actinospores [20]. Therefore, an unexpected characteristic of *P. pseudobranchicola* infections in BC waters was the absence of infection in farmed Atlantic salmon despite the relatively high prevalence in wild Pacific salmon throughout the study area. Approximately 21% (*n* = 199) of the Atlantic salmon in the present study were sampled between 22 and 150 dps. Of these, the 84 salmon that were put to sea between May and October were most likely to have been at immediate risk of infection according to the Norwegian data. The absence of detectable infections in these 84 fish was therefore unlikely to have been because they were sampled prior to exposure or following resolution of the infection. In BC, farmed Atlantic salmon are sentinels for the presence of *K. thyrsites* actinospores in waters adjacent to Vancouver Island [40], suggesting they would also be reliable as sentinels for *P. pseudobranchicola* actinospores, which is supported by the earlier report of 8% prevalence in this species [26]. Farmed Chinook salmon also serve as actinospore sentinels, as reported here and earlier [26]. The absence of clinical parvicapsulosis in BC farmed salmon (Dr. L. Sitter, DFO-FHAIP, Personal Communication), similar to that reported in Norway [15] and in Washington State [12], is further evidence that the epidemiology of *P. pseudobranchicola* in Pacific waters is dissimilar to that in Norway. Specifically, our findings suggest that the epidemiological differences may in part be explained by patterns of actinospore distribution and abundance at or near salmon farms.

A hypothetical framework of parasite transmission may assist in understanding the patterns of *P. pseudobranchicola* infections in farmed and wild salmon in BC. This framework is informed by the distinct spatial and temporal patterns of two relatively well-described myxozoan parasites in the northeast Pacific: *K. thyrsites* and *P. minibicornis* in farmed Atlantic salmon and wild Pacific salmon, respectively. Furthermore, patterns of infection in fish hosts provide insights into the distributions of the invertebrate hosts and the infective actinospores [40]. Knowledge of the variation in the severity of *K. thyrsites* infections among production regions in the vicinity of Vancouver Island is well enough established that farms in these regions have predictably high or low impacts from the infection [41]. In addition, controlled exposure and seawater filtration studies indicate that infection prevalence and the concentration of water-borne *K. thyrsites* DNA is greatest in the summer and autumn [42, 43]. These patterns suggest a transmission scenario (TS-1) in which the actinospores occur in seawater adjacent to Vancouver Island with an abundance that varies seasonally and geographically. Alternatively, infections with *P. minibicornis* are acquired by juvenile and adult Pacific salmon and increase in prevalence and/or severity following salmon migration to the ocean or into the river to spawn [7, 8, 44]. These patterns are consistent with a transmission scenario (TS-2) in which actinospores are restricted to the estuary and/or lower reaches of some salmon natal rivers [13]. Pink salmon and chum salmon juveniles migrate to the ocean between late February and late April and may be exposed to *P. pseudobranchicola* under either TS-1 or TS-2 scenarios. However, our inability to detect the infection in a high proportion of wild salmon caught between May and July tends not to support a TS-2 scenario. Instead, a TS-1 scenario is favoured by the rare to sporadic occurrence of *P. pseudobranchicola* among farmed salmon, combined with the apparent acquisition of infections at sea among wild salmon. The wide geographic distribution of the infection among wild and farmed salmon in Norwegian waters [18–20] also supports a TS-1 scenario. We suggest that the limited impact of *P. pseudobranchicola* on farmed salmon in Pacific waters is due in part to differences in the identity, abundance or local distributions of the invertebrate host relative to Norwegian waters. Framing future research in the context of the TS-1 and TS-2 hypotheses will inform an improved understanding of the spatial and seasonal distributions and abundances of *P. pseudobranchicola* actinospores in coastal BC waters.

Infection with *P. pseudobranchicola* was inconsistently associated with histopathological lesions in the pseudobranch. The association was greatest in Chinook salmon, whereas in chum salmon with higher parasite burdens, the association was weak. In qPCR-uninfected Pacific salmon and in most specimens examined from infected salmon, normal pseudobranch histological morphology was observed, as described previously [12], suggesting that an infection threshold must be exceeded before visible tissue damage occurs. The data further suggest the threshold for parasite-inducted pathology is lower in Chinook salmon relative to chum salmon. While not ruling out a role of other pathogenic stimuli, the pseudobranchitis associated with *P. pseudobranchicola* infections in juvenile wild Pacific salmon was similar to earlier descriptions from farmed Atlantic salmon [15, 20] and from farmed coho salmon infected with *Parvicapsula* sp. [12]. Compartmentalisation of the pseudobranch into damaged and healthy zones in which filaments adjacent to affected areas remained visibly normal as previously reported [20] may indicate a non-uniform initial establishment of the infection, possibly by direct exposure to actinospores from seawater or from the blood-borne stages visualised earlier [20]. The preference of pseudobranch over kidney and gill as the site of infection in Pacific salmon was similar to that reported in Atlantic salmon [15, 20]; however, observations of gross pseudobranch or ocular lesions or of clinical manifestations of the infection were not collected in this study and it is premature to speculate about health consequences of the infections to Pacific salmon. Notable also was the rarity of intense infections which permitted the development of myxospores in the Pacific salmon, suggesting the infections were pre-patent at the time of the autumn surveys because of insufficient development time or thermal history [20]. Alternatively, the rarity of myxospores may indicate host incompatibility among Pacific salmon as suggested from a Norwegian study in which infections without myxospores were detected among rainbow trout farmed in netpens neighboring those with Atlantic salmon presenting with myxospores [19]. A host effect may also explain the involvement of pseudobranch during *P. kabatai* infections in farmed coho salmon [12] but not in pink salmon [3], although the possibility of mixed infections with *P. kabatai* and *P. pseudobranchicola* in the coho salmon cannot be ruled out.

## Conclusions

In waters of the northeast Pacific Ocean, wild Pacific salmon became infected with *P. pseudobranchicola* in their first year at sea, and the prevalence in pink salmon and chum salmon was significantly greater in autumn compared with summer surveys. Myxospores were detected in two Chinook salmon with high parasite burdens. Pseudobranch was the preferred site of infection relative to gills or kidney and in the Pacific salmon, pathological changes in the pseudobranch were similar to those reported in farmed Atlantic salmon in Norway. Although inconsistently associated with the infection, a focal granulomatous pseudobranchitis was observed. Health consequences to Pacific salmon were not assessed. The parasite was detected in farmed Chinook salmon but not in farmed Atlantic salmon. To better understand this epidemiological discrepancy and the seasonal patterns of infection, two transmission scenarios were hypothesised to assist in the characterisation of actinospore distribution in BC waters. Further research should be undertaken to identify the invertebrate host and to describe the life cycle, including myxospore development and resolution of infection among species of Pacific salmon.

## Supplementary Information

**Additional file 1: Table S1.** Weight of wild Pacific salmon (*Oncorhynchus* spp.). **Table S2.** Histopathological scores and in situ hybridisation by ranked qPCR parasite burdens. **Table S3.** Seasonal prevalence of *Parvicapsula pseudobranchicola* in wild salmon by regions. **Table S4.** *Parvicapsula pseudobranchicola* in matched tissues.

## Data Availability

Sequence data reported herein have been deposited in GenBank under accession numbers OP133361, OP133362 and OP133363. Additional datasets will be made available upon reasonable request.
