# Supplementary material for: Parvicapsula pseudobranchicola in the northeast Pacific Ocean is rare in farmed Atlantic salmon Salmo salar despite widespread occurrence and pathology in wild Pacific salmon Oncorhynchus spp
Source: Parasit Vectors. 2023 Apr 21;16:138. doi: 10.1186/s13071-023-05751-y (PMC10122293; doi:10.1186/s13071-023-05751-y)
Supplement: Supplementary file 1 — Additional file 1: Table S1. Weight of wild Pacific salmon (Oncorhynchus spp.). Table S2. Histopathological scores and in situ hybridisation by ranked qPCR parasite burdens. Table S3. Seasonal prevalence of Parvicapsula pseudobranchicola in wild salmon by regions. Table S4. Parvicapsula pseudobranchicola in matched tissues. [file 13071_2023_5751_MOESM1_ESM.pdf]

Table S1. Weight of wild Pacific salmon (*Oncorhynchus* spp.) collected from coastal waters of British Columbia (BC) and from international waters of the Gulf of Alaska

Species	Year	Survey[1]			U-statistic[5]	P-value[5]
		Winter [2]	Summer[3]	Autumn[4]		
Chum	2008		{25}6.9 (4.3 – 8.9)			
	2011		{30}18.0 (14.2 – 19.4)			
	2012		{10}3.7 (2.4 – 6.2)	{11}82.6 (59.3 – 91.0)	0.00	<0.001
	2013		{17}13.7 (8.9 – 20.7)			
	2014		{51}15.1 (12.3 – 22.1)	{8}88.7 (68.2 – 91.5)	5.00	<0.001
	2015		{4}24.8 (22.2 – 28.0)			
	2019		{139}23.0 (18.0 – 32.0)	{165}111.0 (95.0 – 126.0)	22.50	<0.001
	2020	{88}758.5 (337.5 – 1326.0)		{130}131.5 (99.8 – 175.8)		
	2021		{182}23.0 (18.0 – 30.0)	{294}107.0 (87.0 – 130.0)	23.00	<0.001
	Total[6]	88	458	608		
Pink	2010		{60}5.6 (3.4 – 8.8)			
	2012		{30}1.5 (1.1 – 3.2)			
	2014		{50}12.4 (7.5 – 16.3)	{10}67.9 (58.9 – 69.9)	0.00	<0.001
	2019		{6}19.0 (14.3 – 22.0)	{21}74.0 (61.0 – 82.0)	0.00	<0.001
	2020	{9}310.0 (245.5 – 483.0)		{109}107.0 (81.5 – 129.0)		
	2021		{43}16.0 (14.0 – 18.0)	{43}75.0 (53.0 – 89.0)	0.00	<0.001
	Total	9	189	183		
Chinook	2019		{4}26.5 (14.5 – 36.3)			
	2021			{80}81.5 (43.0 – 153.5)		
	Total		4	80		
Coho	2019		{1}56.0			
	2020	{27}788.0 (619.0 – 861.0)				
	Total	27	1			
Sockeye	2019		{8}21.5 (9.3 – 23.5)	{10}31.0 (24.3 – 33.3)	16.00	0.036
	Total		8	10		

[1]{Number weighed} median weight in grams (interquartile range); [2]Gulf of Alaska, March; [3]BC, May to July; [4]BC, September to October. [5]Statistical significance of differences in weight between summer and autumn surveys (Mann-Whitney test). [6]Total number of fish weighed.

Table S2. Semi-quantitative scores of pseudobranch histopathological changes and in-situ hybridization observations in Chinook salmon (*Oncorhynchus tshawytscha*), chum salmon (*O. keta*) and pink salmon (*O. gorbuscha*) by ranked qPCR burdens of *Parvicapsula pseudobranchicola*.

**Species**	**Copies/ngDNA**	**H-path[1]**	**ISH[2]**
Chinook	24.35	+	0
Chinook	26.99	+	+++
Chinook	30.88	++	+
Chinook	34.47	+	+
Chinook	36.41	++	+++
Chinook	37.14	++	0
Chinook	40.36	+	0
Chinook	222.30	++++	++
Chinook	260.95	++++[3]	+++
Chinook	346.50	+++	+++
Chinook	659.03	++[3]	+++
Chum	183.33	+++	+++
Chum	249.62	+	+++
Chum	284.95	+	+
Chum	349.59	+++	+++
Chum	364.40	++	+++
Chum	789.39	++	++
Chum	1505.66	++	+
Chum	3410.98	+	+++
Pink	11.46	++	0
Pink	11.47	++	+
Pink	17.18	+++	+++
Pink	22.71	++	0
Pink	40.93	+++	++
Pink	46.33	++	0
Pink	64.82	++	+++
Pink	71.89	+++	+++
Pink	210.97	++	+++

1. Histopathology scores: +, normal to mild; ++, mild; +++, moderate’ ++++, extensive.
2. In-situ hybridization: 0, no stained parasites per section; +, <5 stained parasites; ++, 6 – 10 stained parasites; +++, >10 stained parasites.
3. Myxospores observed in pseudobranch.

Table S3. Prevalence of *Parvicapsula pseudobranchicola* in pseudobranch of wild pink salmon (*O. gorbuscha*), chum salmon (*Oncorhynchus keta*) and Chinook salmon (*O. tshawytscha*) from summer and autumn surveys in coastal regions of British Columbia.

Year	Region[1]	Pink		Chum		Chinook
		Summer[2]	Autumn[3]	Summer	Autumn	Autumn
2019	DES			0 (10)[4]		
	GIS		25.0 (4)	14.0 (50)	100.0 (23)	
	HOW		100.0 (10)	0 (9)	100.0 (8)	
	JDF		100.0 (1)		79.4 (34)	
	SOG		16.7 (6)	25.7 (70)	100.0 (112)	
	Total[5]		21	139	177	
2021	BUT			0 (10)		
	DES	0 (5)	100.0 (11)	0 (10)	92.6 (27)	60.0 (5)
	DIS	25.0 (20)		10.0 (20)		
	HOW			0 (3)		
	SOG	0 (18)	96.9 (32)	5.8 (139)	97.8 (267)	69.3 (75)
	Total	43	43	182	294	80

[1]BUT, Bute Inlet; DES, Desolation Sound; DIS, Discovery Islands; GIS, Gulf Islands; HOW, Howe Sound; JDF, Strait of Juan de Fuca; SOG, Strait of Georgia. [2]May to July; [3]September and October. [4]Percent infected (number examined). [5]Total number of fish tested.

Table S4. Prevalence and burden of *Parvicapsula pseudobranchicola* in individually matched kidney, gill and pseudobranch samples from wild chum salmon (*Oncorhynchus keta*), pink salmon (*O. gorbuscha*) and chinook salmon (*O. tshawytscha*) collected in the 2021 autumn survey from coastal British Columbia, Canada.

Species	N[1]	Kidney		Gill		Pseudobranch	
		%Inf[2]	Burden[3]	%Inf	Burden	%Inf	Burden
Chum	293	61.1	0.09 (0.04 – 0.21)	75.4	0.29 (0.10 – 0.88)[4]	97.6[5]	18.5 (4.26 – 47.10)[6]
Pink	43	25.6	0.04 (0.02 – 0.13)	62.8	0.14 (0.05 – 0.40)	97.7	2.96 (0.57 – 6.68)
Chinook	80	10.0	0.02 (0.02 – 0.032)	16.3	0.16 (0.06 – 0.37)	68.8	2.90 (0.43 – 10.71)

[1]Number tested; [2]Percent infected; [3]Median qPCR copy number / ngDNA (interquartile range); [4]Significance of burden difference between kidney and gill in chum (U-statistic=10842.0, P<0.001); [5]Signficance of prevalence differences among tissues: chum (X^2=115.8, *df*=2, P<0.001), pink (X^2=47.5, *df*=2, P<0.001), Chinook (X^2=115.8, *df*=2, P<0.001); [6]Significance of burden differences among organs: chum (H-statistic=448.03, *df*=2, P<0.001), pink (H-statistic=47.5, *df*=2, P<0.001), Chinook (H-statistic=27.58, *df*=2, P<0.001).
